# Frequency and severity of globus pharyngeus symptoms in patients undergoing thyroidectomy: a pre-post short term cross-sectional study

**DOI:** 10.1186/s12893-015-0037-x

**Published:** 2015-05-01

**Authors:** Fabrizio Consorti, Rosaria Mancuso, Valentina Mingarelli, Eugenio Pretore, Alfredo Antonaci

**Affiliations:** Department of Surgical Sciences, University “Sapienza” of Rome, Viale del Policlinico, 00161 Roma, Italy

**Keywords:** Globus pharyngeus, Thyroid surgery, Assessment of outcome

## Abstract

**Background:**

Globus pharyngeus is a sensation of a lump or foreign body in the throat, sometimes associated with thyroid diseases and surgery. Previous studies investigated this condition with contradictory results, mainly because not standardized instruments of measure were used. The aim of this study was to evaluate the prevalence and severity of globus pattern symptoms in a population of patients three months after a thyroidectomy, and the reduction or increase of pre-existing symptoms or the onset of new symptoms.

**Methods:**

Ninety-five patients (65 women, 30 men, mean age 56.03 ± 12.45) were assessed for globus pattern symptoms before and three months after thyroid surgery (72 patients: benign goiter, 23 patients: papillary cancer). The Glasgow-Edinburgh Throat Scale (GETS) was translated into Italian and used as a validated instrument of measure of the severity of globus pattern symptoms.

**Results:**

The Italian version of the GETS was reliable (Cronbach alpha = 0.85) and valid. Normative data were used to classify patients into 4 groups of severity. A significant decrease of the mean GETS score was observed at the postoperative assessment (13.02 ± 11.84 vs 8.00 ± 11.26; p < 0.01), but beside symptomatic patients who improved we could observe also two other significant groups of patients: asymptomatic patients who developed symptoms and symptomatic patients who remained symptomatic.

**Conclusions:**

The significant decrease of the mean GETS postoperative score was mainly due to the improvement of strongly symptomatic patients. Two other significant outcomes exist and further studies are needed to understand their pathophysiological mechanism.

## Background

Swallowing disorders are frequent in aged people [1], but they are underestimated both by patients and physicians [2], perhaps because their onset is often slow.

Swallowing is a complex motor reflex requiring coordination among the neurologic system, the oropharynx, and the esophagus. A number of disorders, both benign and malignant, can interfere with this process and globus pharyngeus is a common related symptom. It is defined as the feeling of a lump in the throat, it has uncertain origin and constitutes about 4% of all new ENT referrals [3]. According to Deary [4] 6% of his study group of 1158 women complained about globus and Thompson [5], in a community based study, reported that 45.6% of the general population had experienced globus sensation at some time in their lives. Globus was first described by Purcell in 1707 who coined the term *globus hystericus*, (globus originating from the Latin meaning “ball” and “hystericus” reflecting the supposed psychological component of the disorder). Traditionally patients presenting with globus symptoms were referred to psychiatrists and a study demonstrated that the these subjects were significantly higher on neuroticism and low on extra-version scales and have significantly elevated levels of psychological distress, including anxiety, low mood, and somatic concern when compared with the control subjects [4]. The disorder was renamed globus pharyngeus in 1968 by Malcomson [6] and more recently it has been defined as a persistent or intermittent sensation of a lump or foreign body in the throat for at least 12 weeks with occurrence of the sensation between meals, absence of dysphagia and odynophagia, absence of pathological gastroesophageal reflux, achalasia, or other motility disorder with a recognized pathological basis (e.g., scleroderma of the oesophagus) [7]. Psychological and psychiatric characteristics could be relevant to the experienced discomfort but are unlikely to be etiologically significant. In fact, several potential organic causes for globus pharyngeus were supposed, such as lingual tonsillar hypertrophy, cervical osteophytes, iron deficiency anemia, temporomandibular joint disorder [7]. Laryngopharyngeal reflux [8], hypertensive upper esophageal sphincter [9] and autoimmune diseases [10] have been considered potential causes. Two recent reviews [11,12] summarized the main current findings about globus.

The role of thyroid diseases has also been explored [13]. Goiter is often associated with globus like symptoms, but the exact mechanism is poorly understood, particularly in the absence of a significant retrosternal extension. Burns [14] found that one-third of patients with a thyroid mass complained of globus pattern symptoms. Moreover post-thyroidectomy patients may also complain of globus pattern symptoms, although these symptoms frequently settle with time [15,16]. According to Maung [17] thyroid surgery does not aggravate but rather improves preexisting globus pattern symptoms, while in the study of Wassermann [18] these symptoms increased after surgery. All of these studies relied on the mean value of a score and did not explicitly present data about the rate of asymptomatic patients developing globus pattern symptoms in the post-operative period. The aim of this study is to evaluate the prevalence and severity of globus pattern symptoms in a population of patients three months after thyroidectomy, as a result of reduction or increase of pre-existing symptoms or as the onset of a new set of symptoms.

## Methods

In this cross-sectional longitudinal study, a clinical series of patients operated for thyroid disease were assessed to measure the prevalence and severity of globus symptoms, before and three months after surgery.

The survey was done at the department of Surgical Sciences, recruiting patients candidate to surgery for any thyroid disease from January 2013 to April 2014. Criteria of exclusion were substernal goiter, previous neck surgery, extracapsular cancer, history of gastro-esophageal reflux and poor linguistic skill. This last condition was set because of the use of an instrument of rating of globus symptoms based on a questionnaire in which a rather rich terminology is used. A poor understanding could have hampered the reliability of the assessment. Only patients who underwent a total thyroidectomy were considered.

From a series of 105 patients, 10 met the exclusion criteria, so 95 patients were considered. They were 65 women and 30 men, mean age 56.03 ± 12.45. Indications to surgery were multinodular goiter or cytologically suspected cancer and the final diagnoses were benign goiter in 72 patients and papillary cancer in 23 patiens. All patients routinely underwent pre and postoperative indirect laryngoscopy for vocal cords control and none of the considered patients had vocal cord palsy.

The Glasgow-Edinburgh Throat Scale (GETS) [19] was used to measure the presence and severity of globus symptoms. The GETS is a 10-items questionnaire, based on a 8 grade Likert scale, from 0 (absent) to 7 (unbearable). The total GETS score is computed summing the score of the 10 items, so the highest possible score is 70. Each item explores a different symptom in the domain of globus pharyngeus. In its original form, the GETS showed a good reliability, with a Cronbach alpha value of 0.83. Factor analysis revealed the presence of three subscales, grouping set of items: related to swallowing, related to globus sensation, related to painful throat. Two more items are present, whose score is not computed in the total GETS score but accounts for the somatic distress reaction to symptoms (SDR score: how much time do you spend thinking about your throat? At present, how annoying do you find your throat sensation?). We translated the 12 items into Italian and tested the instrument in a preliminary set of 30 patients, different from those recruited for the present study. This set was also used to train the two researchers in charge of doing the interviews. All the interviews in the study were done by one of the two trained researchers (RM or EP). All patients gave their verbal consent to the interview. The ethical committee of the department stated that ethical approval was not needed because the filled form of the interview was included in the medical record as a special part of the clinical history section.

### Statistical methods

To define the study size, from previous literature [13,14,16,18] we considered the hypothesis that about 50% of patients who were symptomatic before the operation would retain some symptoms, while 5% of asymptomatic patients will develop symptoms in the postoperative period. With an alpha error of 5% and a power of 90%, the sample size needed to detect as significant the variation of the presence and severity of the symptoms in the postoperative period was constituted by 50 symptomatic and 15 asymptomatic patients.

The re-validation of the Italian version of the GETS was performed with classical item analysis, computing the Cronbach alpha index. A principal component factor analysis was also performed, with varimax rotation and extracting components with an eigenvalue > 1.

Differences in proportion were assessed by chi square test, differences in the GETS and SDR scores by Wilcoxon test for non-parametric data. All calculations were done with SPSS™ ver.20 statistical software.

## Results

### Validation of the Italian version of the GETS and normative classification of the score

The Cronbach alpha value for the 10-items GETS scale was 0.85 and 0.92 for the two items on distress reaction. Factor analysis for the 10 items of the GETS demonstrated the same three sub-factors as in Deary’s study [19] (Table 1).

**Table 1:** **Rotated component matrix of the loading factors for the 10 items of GETS**

**Items**	**Component**		
	**1**	**2**	**3**
Feeling of something stuck in the throat	.448	**.647**	.035
Pain in the throat	.141	.075	**.905**
Discomfort/irritation in the throat	.330	**.597**	.362
Difficulty in swallowing food	**.841**	.133	-.146
Swelling in the throat	-.076	**.724**	.192
Throat closing off	**.658**	.494	.187
Catarrh down throat	.072	**.607**	-.254
Can’t empty throat when swallowing	**.760**	.103	.291
Want to swallow all the time	.458	**.505**	.149
Food sticking when swallowing	**.874**	.058	.140

Component 1: symptoms related to swallowing; Component 2: symptoms related to globus sensation; Component 3: pain.

Factor loadings represent how much a factor explains a variable in factor analysis. It is accepted that a value higher than .50 indicates that the answer to that particular item is mainly influenced by that factor. Loadings > .50 are highlighted in bold.

To obtain an outcome more clinically oriented than a score without thresholds, we divided the distribution of the GETS score into five classes, setting thresholds at the 20^th^ (range:0÷2 points), 40^th^ (range:3÷8 points), 60^th^ (range: 9÷12 points), 80^th^ (range:9÷20 points) and over the 80^th^ centile (>20 points) of the preoperative distribution of scores. This method of classification, developed for educational research, proved to be useful in analyzing the distribution of a rank of scores [20]. According to these thresholds, we classified patients as asymptomatic, mildly symptomatic, symptomatic (3^rd^ and 4^th^ class together), strongly symptomatic .

Table 2 reports the synthetic data of preoperative assessment for the 12 items.

**Table 2:** **Synthetic data for the 10 items of GETS and the 2 items on somatic distress reaction, preoperatively assessed on 95 patients**

	**Glasgow-Edinburgh Throat Scale (GETS): 10 items**										**Somatic distress reaction**	
	**stuck**	pain	**irritation**	difficult swallowing	swelling	closing off	**catarrh**	can’t empty	**swallow all time**	food sticking	time	find annoying
Mean score	2.27	.66	1.66	.97	1.07	1.31	1.52	1.04	1.48	1.03	1.59	1.85
St.dev.	1.98	1.26	2.00	1.63	1.80	2.06	2.13	1.66	2.21	1.76	2.18	2.24
N.of score 0	27	68	46	62	61	57	51	57	54	62	50	41
N.of score 7	3	1	2	1	2	3	6	1	6	1	6	7

In bold the most frequent four symptoms.

### Pre-postoperative comparison of GETS and SDR scores

The mean GETS preoperative score was 13.02 ± 11.84 (C.I. 95%: 10.64 ÷ 15.40, min = 0; max = 57), the mean postoperative score was 8.00 ± 11.26 (C.I. 95%: 6.01 ÷ 11.59, min = 0; max = 51). This difference was significant at Wilcoxon rank sum test (p = 0.005). According to the classification of scores, 38.9% of patients in the postoperative period were asymptomatic (37 pts, GETS score 0÷2), 23.1% were mildly symptomatic (22 pts, 3÷8), 30.5% were symptomatic (29 pts, 9÷20) and 7.4% were strongly symptomatic (7 pts, >20) (Table 3). To describe the changes of the GETS score of patients from preoperative to postoperative assessment, we considered the possible main transitions: symptomatic patients who became asymptomatic or less symptomatic and vice versa. Table 4 summarizes these transitions.

**Table 3:** **Distribution of patients in the four classes of GETS score before and after the operation: number of patients (%)**

**Class of GETS score**	**Preoperative**	**Postoperative**
Asymptomatic (0-2)	20 (21.1)	37 (38.9)
Mildly symptomatic (3-8)	20 (21.1)	22 (23.1)
Symptomatic (9-20)	36 (37.8)	29 (30.5)
Strongly symptomatic (>20)	19 (20.0)	7 (7.5)
Total	95 (100)	95 (100)

chi square test: p < 0.01.

**Table 4:** **Modification of the level of symptoms, according to GETS score, after the operation, stratified for the four classes of score**

**Class of GETS preoperative score**	**Improved**	**Unchanged**	**Worsened**	**Total**
Asymptomatic (0-2)	0 (0)	16 (80)	4 (20)	20 (100)
Mildly symptomatic (3-8)	4 (20)	8 (40)	8 (40)	20 (100)
Symptomatic (9-20)	21 (58.3)	15 (41.7)	0 (0)	36 (100)
Strongly symptomatic (>20)	16 (84.2)	3 (15.8)	0 (0)	19 (100)

The considered outcome are: improved (moved to a lower class), unchanged, worsened (moved to a higher class). In each cell: number of patients (%).

chi square test: p < 0.01.

The pre and postoperative mean score of the two items for somatic distress reaction was respectively 3.44 ± 4.28 (C.I. 95%: 1.06 ÷ 5.82, min 0, max 14) and 2.43 ± 3.28 (C.I. 95%: 1.77 ÷ 3.09, min 0, max 13). This difference was not statistically significant. Both the pre and postoperative distress scores were correlated with the GETS scores (correlation coefficient for preoperative scores: 0.50; for postoperative scores: 0.76).

## Discussion

Our study confirmed that a relevant proportion of patients with thyroid disease, if carefully enquired with a standardized instrument, complains of globus pattern symptoms of moderate or strong severity (57.8%). A second group declares mild symptoms (21.1%) and only about 20% of patients are completely asymptomatic or just report faint feelings in the domain of globus. When the mean GETS score was considered, a significant decrease of the severity of symptoms was observed after thyroidectomy, but this decrease was mainly due to the improvement of strongly symptomatic patients (Table 3). When the change of the pre-postoperative GETS score of each patient was considered (Table 4), a group of patients appeared who were asymptomatic or mildly symptomatic at the preoperative assessment and whose severity of symptoms increased in the postoperative period (12/95 patients = 12.6%). Another larger group of patients (26/95 patients = 27.4%) were mildly o definitely symptomatic and maintained unchanged their symptoms while the largest group of symptomatic patients improved (37/95 patients = 38.9%). These data indicate that surgery improves the condition of patients with moderate or intense globus pattern symptoms, but may make symptoms appear in asymptomatic of weakly symptomatic patients.

These findings suggest that the pathophysiology of globus pattern symptoms may be different: in some cases connected with thyroid disease, in other cases with the effect of surgery itself. Finally, in some patients of this series, globus pattern symptoms remained unchanged and seemed to be independent both from thyroid disease and from thyroidectomy. An alternative interpretation for the group of patients who remained symptomatic is that thyroidectomy produced the same pathophysiological effect as the presence of an ill thyroid did before the operation.

Other studies enquired the relationship between thyroid surgery and globus pattern symptoms, but all of them had limits either in the representation of data or in the extent and method used to assess globus pattern symptoms. In a small scale study, Maung et al. [17] did not find any significant worsening of GETS score three and twelve months after surgery. Some of the items of the GETS showed instead an improvement. Unfortunately the authors did not provide the mean total GETS score nor the mean values for each item, so a comparison with our study is not possible. Burns et al. [ 14 ] in a series of 200 patients who underwent thyroidectomy found 58 patients (29%) with preoperative globus symptoms, which reduced to 12 (6%) 3-6 months after the operation. The authors used a visual-analogue scale graduated from 1 to 10 to let the patient self-assess the globus sensation as a whole. Wasserman et al. [18] reported instead an increase of globus sensation, from 57.6% to 75.8% of patients one week after thyroid surgery. The focus of their study was on the correlation of symptoms with the function of the internal branch of the superior laryngeal nerve (SLN), which brings sensitivity to the larynx, but they could not find any significant decrease in SLN functioning. Lombardi et al. [16] reported an increase in swallowing alterations after thyroidectomy. They measured the symptoms with an original questionnaire, reporting a very low preoperative score, a strong rise one week after surgery and a progressive decrease after one and three months. Unfortunately they questionnaire fully covered only the dimension of dysphagia and partly the dimension of globus sensation of the GETS. In a wide Italian multicentric study on postoperative complications in 14,943 operations on thyroid, dysphagia was the only symptom considered in the globus pattern domain and only in relation to SLN damage [21]. Finally, Smith-Hammond et al. reported a significant increase of dysphagia – measured with a standardized questionnaire – after spine surgery with anterior cervical approach [22], accessing the same anatomical space as in thyroid surgery.

The terms in which the globus symptoms are described by patients vary largely and this is why we preferably referred to a pattern of globus symptoms. To manage this variability, it is important to rely on a validated and comprehensive instrument like the GETS. The Italian version of the GETS we used had a good reliability and the same factorial structure as the original one. Normative data on the general [23] and ENT population [4] are available and indicate that a significant proportion of people without any evident structural pathology has a score of 1 or 2 at one of the items of the GETS (up to 55.2% for “Catarrh down throat” in [23]). The four classes of score we proposed are coherent with the above mentioned normative data and are clinically oriented. The classification of patients allowed to uncover three different dynamics, which were otherwise obscured by the synthetic presentation of data only by the pre and postoperative means: symptomatic patients who improved their symptoms after surgery, asymptomatic patients who developed symptoms after surgery and patients whose symptoms seemed not to be influenced by surgery.

The main limit of this study is the short follow up interval, limited to three months. It is known that symptoms tend to resolve after one year [24]. Nevertheless, even if symptoms are temporary, it is important to know their frequency in order to make patients informed and aware. Another possible limitation is that each interview was made only by one of the two trained observers, even if they had reached a good inter-rater agreement during the testing phase. We did not assess in any formal way the presence of gastro-esophageal reflux apart from history taking, so we cannot exclude that some of the patients who remained symptomatic could have been suffering from an unknown reflux [25]. We did not correlate symptoms with smoking habit or with other possible risk factors for globus pattern symptoms, such as thyroid volume, since the goal of this study was to measure frequency and severity of pre e and postoperative symptoms in a population of patients operated for thyroid disease.

As a last remark, it is known that a repetitive strain injury of the paralaryngeal muscles can cause globus-like symptoms. This mechanism could be considered either in the event of preoperative or postoperative symptoms. In these cases, manual therapy proved to be effective in quickly relieving the symptoms [26,27].

## Conclusion

In conclusion, research on outcome after surgery must be supported by valid and reliable instruments, especially when outcomes related to symptoms and quality of life are addressed. We demonstrated three possible outcomes after thyroid surgery as to globus pattern symptoms and on this basis further studies are needed to understand the pathophysiological mechanisms leading to these outcomes.
